# Predicting a change in the order of spring phenology in temperate forests

**DOI:** 10.1111/gcb.12896

**Published:** 2015-03-02

**Authors:** Adrian M.I. Roberts, Christine Tansey, Richard J. Smithers, Albert B. Phillimore

**Affiliations:** ^1^ Biomathematics & Statistics ScotlandJames Clerk Maxwell BuildingKing's BuildingsEdinburghEH9 3JZUK; ^2^ Institute for Evolutionary BiologyUniversity of EdinburghKing's BuildingsEdinburghEH9 3FLUK; ^3^ The Woodland TrustKempton WayGranthamNG31 6LLUK; ^4^ Ricardo‐AEAGemini BuildingFermi AvenueHarwellOxonOX11 0QRUK

**Keywords:** chilling, climate change, forcing, growing degree‐day, phenology, plasticity, prediction, shade, species interactions

## Abstract

The rise in spring temperatures over the past half‐century has led to advances in the phenology of many nontropical plants and animals. As species and populations differ in their phenological responses to temperature, an increase in temperatures has the potential to alter timing‐dependent species interactions. One species‐interaction that may be affected is the competition for light in deciduous forests, where early vernal species have a narrow window of opportunity for growth before late spring species cast shade. Here we consider the Marsham phenology time series of first leafing dates of thirteen tree species and flowering dates of one ground flora species, which spans two centuries. The exceptional length of this time series permits a rare comparison of the statistical support for parameter‐rich regression and mechanistic thermal sensitivity phenology models. While mechanistic models perform best in the majority of cases, both they and the regression models provide remarkably consistent insights into the relative sensitivity of each species to forcing and chilling effects. All species are sensitive to spring forcing, but we also find that vernal and northern European species are responsive to cold temperatures in the previous autumn. Whether this sensitivity reflects a chilling requirement or a delaying of dormancy remains to be tested. We then apply the models to projected future temperature data under a fossil fuel intensive emissions scenario and predict that while some species will advance substantially others will advance by less and may even be delayed due to a rise in autumn and winter temperatures. Considering the projected responses of all fourteen species, we anticipate a change in the order of spring events, which may lead to changes in competitive advantage for light with potential implications for the composition of temperate forests.

## Introduction

Phenology, the timing of recurrent life‐history events, such as leafing, flowering, migration, and reproduction, determines the abiotic conditions and species interactions to which an individual is exposed. In temperate regions the spring phenology of many species correlates negatively with temperature (Roy & Sparks, 2000; Fitter & Fitter, 2002) and has advanced as temperatures have risen in recent decades (Parmesan, 2007). As species vary in their phenological responses to temperature, a change in climate may cause a change in the phenology of one species relative to others in the same community and this may impact on the fitness of one or both species (Visser & Both, 2005; Elzinga *et al*., 2007). The effects of climate change on phenological mismatches between consumers and their resources (Durant *et al*., 2007; Thackeray *et al*., 2010) or plants and their pollinators (Hegland *et al*., 2009) have received substantial attention. In comparison, the potential for climate‐induced changes in phenology to impact on interspecific competition has been relatively overlooked.

Light is a limiting resource in forests over which plants compete. The phenology of different plants in a temperate deciduous forest follows a characteristic chronology, beginning with vernal shade‐intolerant ground flora, and progressing through trees in the understory to those in the canopy (Salisbury, 1921; Rathcke & Lacey, 1985). Leafing phenology directly influences the amount of light penetrating the canopy (Anderson, 1964), which can be a limiting factor on the rate of growth and reproduction in the ground flora (Whigham, 2004). Shade‐intolerant species that rely on the high irradiance levels before canopy closure to flower and fruit may set seed less successfully in advanced shade (Kudo *et al*., 2008). For woody understory species, early leafing prior to canopy development provides opportunities for photosynthesis that partially offset the reductions in photosynthesis once shading has developed (Augspurger *et al*., 2005). As a consequence, if climate change alters the relative phenology of different forest plant species, this may shift the fitness of one species relative to another and the species composition of a forest (Kramer *et al*., 2000).

Accurate predictions of species’ phenology under projected future climatic conditions rely on identifying the relevant cue(s) and the response(s) they elicit. For temperate regions, we know that tree leafing and plant flowering of most species is sensitive to thermal forcing, whereby elevated spring temperatures result in faster development and earlier phenology (Fitter *et al*., 1995; Polgar & Primack, 2011). Some plant species are also sensitive to chilling, whereby lower temperatures during the preceding autumn and winter are associated with advanced phenology (Murray *et al*., 1989; Fitter *et al*., 1995; Yu *et al*., 2010; Polgar & Primack, 2011). A recent cross‐species comparison of the effect of chilling treatments on twigs revealed substantial variation among species in the sensitivity of their phenology to chilling, with canopy species requiring the longest chilling periods to break dormancy (Laube *et al*., 2014). As a consequence of interspecific variation in the thermal sensitivity of phenology, a rise in temperatures may lead to phenological advances in some species and delays for others (Cook *et al*., 2012; Laube *et al*., 2014).

Statistical analysis of the relationship between ambient temperatures and phenological observations represents a major source of insight into cues and sensitivity (e.g., Cook *et al*., 2012). Statistical models fall into two broad classes: (i) regression based, wherein the effect of daily or aggregated temperatures and phenology is estimated and model parameters do not directly relate to known biological processes; (ii) mechanistic, wherein models are constructed to relate to biological processes that have been inferred from experiments, such as the accumulation of growing degree‐days and chilling requirements. Both types of model can become parameter rich, so that long‐time series are required for accurate parameter estimation and informative model comparisons. One of the most exceptional phenological time series is the Marsham record; Robert Marsham began monitoring plant and animal phenology in 1736 and reported his findings to the Royal Society in 1789 (Marsham, 1789). After his death in 1797 his descendants continued recording these events until 1958 (Sparks & Carey, 1995), making this one of the longest phenological time series worldwide. Observations are of first events from around Stratton Strawless Hall in Norfolk, UK (lat = 52.74, lon = 1.29) and in some cases from elsewhere across southeastern England and include the first leafing dates of thirteen tree species, as well as flowering dates of plants and various animal records (Margary, 1926; Sparks & Carey, 1995). Sparks & Carey (1995) examined the thermal sensitivity of these records via application of stepwise regression to monthly temperature averages. In addition to identifying a strong effect of spring forcing on all species, for some species warm temperatures in the preceding autumn were found to correlate with later phenology. In this article, we revisit some of these data with a variety of powerful correlation‐ and mechanism‐based statistical approaches that can be applied to daily temperature data for the inference of thermal cues and the phenological response they elicit (e.g., Chuine, 2000; Roberts, 2008).

In this study, we consider the first leafing and flowering dates of fourteen forest species from the Marsham record. We have two main aims: first, to identify species sensitivities to both spring forcing and autumn/winter chilling; second, to predict how the phenology of species will shift relative to the phenology of other species in the community under a projected climate change scenario. A secondary focus of our work is a comparison of the performance and insights obtained from regression‐based and mechanistic statistical models that seek to explain phenological thermal sensitivity.

## Materials and methods

We focus on fourteen forest plant events from the Marsham time series, which spans the period 1753–1947. Thirteen events were tree first leafing, and one was of wood anemone (*Anemone nemorosa*) first flowering (see Table 1). For further details on this exceptional dataset we refer the reader to earlier works (Margary, 1926; Sparks & Carey, 1995). We excluded the 1938 sycamore (*Acer pseudoplantnus*) record that Sparks and Carey identified as an extreme outlier and potentially erroneous. We matched observations with daily temperatures from the Central England temperature (CET) record, beginning in 1772 (Parker *et al*., 1992). While the Marsham Estate falls outside the triangle of weather stations used to obtain this record, for the period 1960–2009 the daily CET show an excellent correspondence (Pearson's correlation across all days = 0.96, Pearson's correlation per day = 0.67–0.97, with the Marsham location 0.13 °C warmer on average) with daily mean temperatures interpolated to the Marsham location from > 500 UK weather stations (Perry *et al*., 2009). Using CET data will inevitably introduce additional measurement error, which is expected to reduce the explanatory power of our models.

**Table 1:** Summary statistics and model comparisons using Δ AIC (difference in Akaike Information Criterion from best model). Models within 2 units of the best are underlined

Species^a^	Number of years	Mean day of event [ordinal date]	Standard deviation	Δ AIC						
				Null	UniForc	UniChill 1 Sept	UniChill 1 Nov	Time window	Double time window	PSR
hawthorn – *Crataegus monogyna*	143	9 March [67.6]	19.1	119.4	23.7	0.0	25.4	32.5	17.9	15.9
wood anemone – *Anemone nemorosa*	140	25 March [83.8]	13.0	100.3	11.4	2.1	0.1	9.4	6.2	0.0
sycamore – *Acer pseudoplantanus*	134	1 April [91.0]	13.3	66.2	7.4	1.9	12.3	8.6	0.0	8.2
horse chestnut – *Aesculus hippocastanum*	142	4 April [93.8]	10.4	94.7	6.6	0.0	6.5	7.4	0.3	12.7
elm – *Ulmus* (*procera*?)	118	5 April [95.3]	14.9	41.8	0.0	0.4	0.3	4.0	4.3	8.4
birch – *Betula* (*pendula*?)	140	6 April [95.6]	12.9	88.3	18.1	0.0	19.4	22.4	3.8	8.5
rowan – *Sorbus acuparia*	138	6 April [96.0]	11.4	153.2	25.0	0.0	15.1	42.3	17.4	17.3
hornbeam – *Carpinus betulus*	137	7 April [97.5]	15.2	48.5	7.3	0.0	3.9	9.2	8.6	7.5
lime – *Tilia* spp.	140	13 April [102.7]	11.6	125.2	10.7	0.0	3.5	24.6	10.5	13.6
maple – *Acer* (*campestre*?)	96	19 April [108.5]	13.4	38.6	13.8	7.1	14.3	11.4	0.0	7.8
sweet chestnut – *Castanea sativa*	134	19 April [108.6]	11.3	111.1	1.1	0.0	5.1	19.4	12.6	16.1
beech – *Fagus sylvatica*	143	20 April [110.0]	7.8	92.5	0.0	1.0	4.5	10.9	12.0	15.8
oak – *Quercus* spp.	141	23 April [113.1]	10.7	197.9	19.4	2.6	0.0	58.8	42.5	31.8
ash – *Fraxinus excelsior*	129	29 April [118.7]	11.1	53.4	0.0	4.4	0.9	9.1	9.4	15.2

a Species identities follow Sparks & Carey (1995). Latin binomials in parentheses indicate records for which the species is uncertain.

We applied both regression and mechanistic approaches to model the effect of daily temperatures on the Marsham phenological record. The three regression methods that we considered were single and double sliding time‐window regression and *P*‐spline signal regression (PSR). Sliding time‐window regression (Husby *et al*., 2010; Phillimore *et al*., 2012) identifies the period or periods of consecutive days for which the mean temperature best predicts the phenological response. The model is then a linear regression. For the single time‐window potential covariates were daily temperatures from 1 June of the year preceding the event up to the ordinal day (i.e., days from Jan 1st) of the last recorded event. We allowed the duration of the time window to vary from 2 to 120 days and identified the single most predictive time window on the basis of R^2^. This meant that for each species we considered hundreds of possible time‐windows. For the double time‐window analysis we included the most predictive time‐window from the above analysis and average temperature during an earlier time window (start date from June 1st of the previous year and duration 10–120 days) in a multiple regression. We iteratively searched for the time window that yielded the highest R^2^. Throughout we used the Akaike Information Criterion (AIC) to compare model types (Rathcke & Lacey, 1985). In calculating AIC for these models, we included start date and duration of time windows as additional model parameters.

*P*‐spline signal regression allows regression on all daily temperature covariates under consideration (Marx & Eilers, 1999; Roberts, 2008), and we focused on the period from 1 June of the year preceding the event up to the Julian day of the last recorded event. PSR copes with multicollinearity of daily temperatures by smoothing regression coefficients over the time sequence. This is achieved by penalizing differences between coefficients for consecutive days. To cope with the large number of covariates, PSR includes a data‐reduction step through transformation to a smooth *B*‐spline basis and requires estimation of the optimal smoothing parameter through cross‐validation. We used the mgcv package (Wood, 2001) in R (R Development Core Team, 2014), and set the degree of differences and order of *B*‐splines as advised in Roberts, (2012). The degree of complexity of the fitted curve is expressed by the effective degrees of freedom.

Mechanistic models for phenology can be traced back to the 18th Century (Réaumur, 1735) and are based on the idea that the rate of physiological development depends on the accumulation of daily temperatures or thermal time. Here we have chosen to use two models, UniForc (Hänninen, 1990; Chuine, 2000) and UniChill (Chuine, 2000). UniForc is the simpler of the two. This predicts that the phenological event occurs once sufficient forcing units, F*, have been accumulated. The forcing function, *R*_*f*_, is given by

$$R_{f}\left(x_{t}\right)=\frac{1}{1+e^{b_{f}\left(x_{t}-c_{f}\right)}},$$

where *x*_*t*_ is the temperature on day *t*, and *b*_*f*_ < 0 and *c*_*f*_ > 0 are parameters to be estimated. So the event is predicted to occur on the first day *t*_*b*_ such that

$$\sum_{t=t_{1}}^{t_{b}}R_{f}\left(x_{t}\right)\geq F^{*},$$

where *t*_1_ is the day when forcing starts, which is also to be estimated, resulting in a total of four parameters.

The UniChill model extends the UniForc model by adding a chilling requirement to the forcing criterion. It is a sequential arrangement where forcing only starts once sufficient chilling units, C*, have been accumulated. So *t*_1_ is set such that

$$\sum_{t=t_{0}}^{t_{1}}R_{c}\left(x_{t}\right)\geq C^{*},$$

where *t*_0_ is the date that the chilling process starts and the chilling function, *R*_*c*_, is given by the more flexible function

$$R_{c}\left(x_{t}\right)=\frac{1}{1+e^{a_{c}{\left(x_{t}-c_{c}\right)}^{2}+b_{c}\left(x_{t}-c_{c}\right)}},$$

with *a*_*c*_, *b*_*c*_ *,* and *c*_*c*_ are parameters to be estimated. As in (Chuine, 2000), we fix *t*_0_ to either 1 September or 1 November in the year preceding the event, rather than estimating it. As such, the UniChill model has seven parameters to be estimated.

We fitted the mechanistic models to the data using heuristic optimization algorithms that sought to minimize the root mean square error between the predicted and observed phenology. This proved challenging due to the inherent discreteness of the objective function, having multiple minima and a degree of parameter redundancy. We attempted to improve this by making the predicted response continuous through linear interpolation. We used two sets of algorithms to ensure good solutions were found (a) simulated annealing with the GenSA package (Xiang *et al*., 2013) in R, starting from 200 start points and (b) particle swarm optimization (PSO) using the hydroPSO package (Zambrano‐Bigiarini & Rojas, 2013) in R. Both required sensible setting of initial values and parameter ranges (e.g., through use of UniForc estimates for estimating the UniChill model).

To project future phenology, future temperature projections were required. We followed the UK Climate Impacts Programme (UKCP09) weather generator approach (Jones *et al*., 2009), treating the Central England temperature in the period 1961–1990 as a temperature baseline. For the 25km grid square that contains 52.74°N, 1.29°E, we obtained 1000 samples of the posterior distribution of projected change in monthly temperatures for 2010–2039 and 2040–2069 under a fossil fuel intensive SRES scenario (A1F1) and allowing for random sampling of model variants. We then added these projected changes to the baseline temperatures to get 1000 thirty‐year time series for each time period, capturing both year‐to‐year variability and uncertainty in the temperature projection.

To encompass parameter uncertainty in predictions based on the mechanistic models, we put them into a Bayesian framework, which enabled us to generate samples from the joint posterior distribution of the parameters using Monte Carlo Markov chain (MCMC). For each species, we selected the best fitting model. Again we chose to use a continuous form of the predicted response through linear interpolation. The models were simplified to reduce mixing problems caused by parameter redundancy. The parameter *c*_*f*_ was fixed to the maximum likelihood estimate for both UniForc and UniChill models. For the UniChill model the parameter *c*_*c*_ was also fixed, and *b*_*c*_ was constrained to be positive. We selected weakly informative priors for parameters. Convergence and mixing were assessed by Geweke's (1992) and Heidelberger & Welch's (1983) convergence diagnostics for single chains along with Gelman and Rubin's convergence diagnostic (Gelman & Rubin, 1992) on four parallel chains. For the UniForc model, burn‐in periods of 10 000 iterations were followed by a minimum of 50 000 iterations thinned to a sample of 1000. For the UniChill model, the burn‐in period employed was 50 000 iterations, which followed by a minimum of 200 000 iterations and then thinned to a sample of 1000. Where indicated by convergence diagnostics, we ran the chains longer. We used JAGS MCMC software package (Plummer, 2003), along with the Coda packages in R.

We then applied each sample of the phenology model parameter posterior distribution to a different sample of the projected time series, giving us 1000 samples of a 29‐year projected phenology time series. We used this distribution to compare the relative phenology of different species pairs.

As a test of our predictions, we assessed the impact of recent temperature changes on the relative timings of first leafing of two tree species during the period 1999–2011. We based this analysis on 704 silver birch and 558 pedunculate oak records that citizen scientists have contributed to the UK Phenology Network (www.naturescalendar.co.uk) from locations within 1° latitude and 1° longitude of Stratton Strawless Hall (52.74°N, 1.29°E). Note that we do not know the species identity of the oak and birch recorded by the Marsham family (Sparks & Carey, 1995). We assessed the average annual difference in phenology in a mixed effects model (Bates *et al*., 2012), treating phenology as a response, year as a random effect and species as a fixed effect.

Except where stated otherwise, statistical analyses were conducted using R (R Development Core Team, 2014).

## Results

### Thermal cues

**Figure 1:**
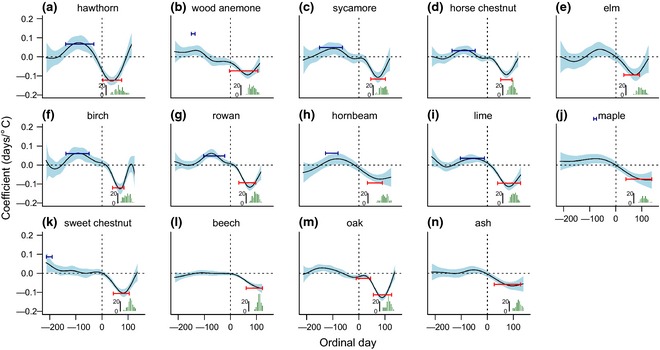
Predicted coefficients (black line) from *P*‐spline signal regression model (see Materials and methods) for the effect of daily temperatures during the preceding and current year on phenology of the fourteen species (a‐n). Ordinal dates start on Jan 1st in the year of the event and ordinal dates with a value <1 refer to the previous year. The light blue region indicates 95% approximate confidence intervals on individual coefficients. Histograms present the temporal distribution of observations for each event in the Marsham record. The red (forcing) and blue (chilling) horizontal bar identify the time period(s) identified using the sliding‐window approach, with the bar position on the y axis = average coefficient over the time window.

Time‐window and PSR models explain 29–73% of the interannual variation in phenology (Table S1a‐c) and identify highly congruent temperature‐forcing periods that start a month or more before the first event and overlap with the distribution of events (Fig. 1). Sensitivity to forcing during the best time‐window ranges from −5.06 days °C^−1^ in beech to −9.33 days °C^−1^ in hawthorn (Table S1a).

The single time‐window is outperformed by the double time‐window and/or PSR model for all species other than elm, beech, and ash (Table 1). In most cases double time‐window and PSR models identify coincident periods of chilling sensitivity in the latter part of the preceding year (Fig. 1). This suggests that warmer conditions in the autumn–winter period have a delaying effect on phenology (Fig. 1). The importance of chilling varies between species, being most extreme for hawthorn and birch, with chilling slope estimates of 7.36 and 5.39 days °C^−1^, respectively (Table S1a). Oak behaves differently in the double time‐window analysis in that the first window is identified as playing a forcing rather than chilling role (Fig. 1m, Table S1b).

**Figure 2:**
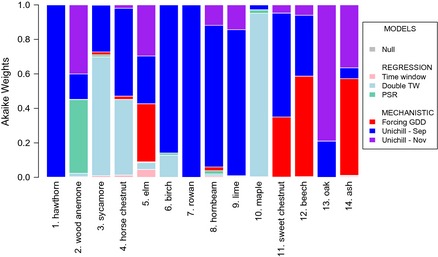
Akaike weights comparing all models for each species.

Mechanistic models, based on growing degree‐days, outperform the regression models for most species, the exceptions being wood anemone, sycamore, horse chestnut, and maple (Fig. 2, Table 1). However, the insights from double time‐window and PSR models broadly agree with those gained from mechanistic models, demonstrating the utility of such straightforward correlative approaches for identifying thermal cues.

The forcing‐only model (UniForc) outperforms the chilling and forcing (UniChill) model for first leafing of elm, beech, and ash. Where the UniChill model performs best, September 1st is the preferred UniChill start date for all species except oak, where November 1st is preferred. For most species the chilling function means that only days where temperatures are below a threshold varying from 10 to 17 °C contribute to chilling (Fig. S1, Table S1b). However, in the case of horse chestnut and oak the chilling function unexpectedly exhibits a trough shape and for wood anemone there is a positive relationship between temperature and the corresponding chilling units (Fig. S1). We find no tendency for later spring species to have larger chilling requirements, as captured by C* (Fig. S1, Table S1e & f). Where one of the UniChill models is preferred, we find that the mean date of the chilling requirement being met is broadly coincident with the start date for forcing under the UniForc model, but that the standard deviation of this date among years can be substantial, for example, for birch = 6.36 days (Table S1 d‐f). With the exception of beech and ash, forcing functions are sigmoid over the relevant temperature range. Species with early phenology accumulate more forcing units at lower temperatures than species with later phenology (Fig. S1).

There was evidence for a degree of first‐order temporal autocorrelation in the model residuals for some species, in particular hornbeam. This may arise from a carryover between one year and the next, but could equally be due to autocorrelation in recorder behavior or weather. Consequently, we will have slightly underestimated parameter uncertainty.

A striking finding to emerge from this study is the early timing of the chilling period for those species where such an effect was supported (Fig. 1). In the PSR model significantly positive coefficients extend back to about 122 days into the previous year (September 1st), in agreement with a general preference for September 1st as the UniChill model start date. For oak, high temperatures as far back as the preceding summer months appear to delay spring phenology (Fig. 1), as Sparks & Carey (1995) noted.

### Phenology prediction

**Figure 3:**
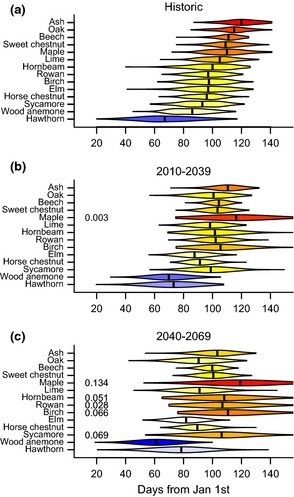
Violin plots of the distribution of spring events in (a) the Marsham dataset and projected for (b) 2010–2039 and (c) 2040–2069. Projections are based on the mechanistic model with the lowest AIC value. They capture modeled uncertainty both in temperature change and phenology model parameters, as well as baseline levels of year‐to‐year variation in temperature (see Materials and methods). Numeric values to the left of violins report the proportion of projections that resulted in no event by ordinal day 250. Median (excluding cases where no event was predicted) phenology is shown as a black vertical line. Colors correspond to the position of each species’ median on a gradient from early (blue) to late (red).

When we predict future phenology on the basis of projected temperatures under a fossil fuel intensive SRES scenario (A1F1) for 2010–2039 and 2040–2069 we find that the median first dates of all species are shifted relative to historic values (Fig. 3). Several species with late spring phenology, sweet chestnut, oak, beech, and ash, are predicted to advance their phenology considerably. For instance, by 2010–2039 the predicted median oak first leafing date is 14.3 days earlier than the historic records and by 2040–2069 it is another 10.3 days earlier. In comparison, several of the species with early spring phenology, especially those that are highly sensitive to chilling, such as hawthorn and birch, are predicted to be delayed or advance less. In addition, we find that for both projected periods the chilling requirements of some species will not be met in years with especially warm conditions (Fig. 3b,c), mirroring the findings of a similar projection of North American tree phenology (Morin *et al*., 2009).

**Figure 4:**
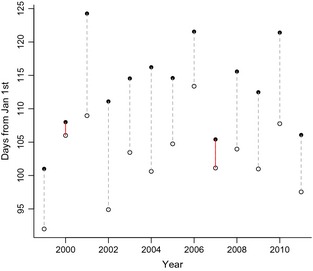
The annual means for the first leafing dates of silver birch (empty circles) and pedunculate oak (filled circles) based on UK Phenology Network data. The red lines denote the 2 years for which the Central England temperatures from the previous September 1st to end of year were highest.

At the community level, the species’ responses are predicted to result in increased synchrony of spring phenological events by 2010–2039, and a re‐arrangement of the timing of events by 2040–2069 (Fig. 3). This chronological shuffling is most apparent if we consider phenology and predictions for species in a pairwise fashion (Table S2). If we take birch and oak as an example: in the Marsham dataset birch came into leaf before oak in >90% of years, by 2010–2039 this is predicted to decrease to 38% of years and by 2040–2069 oak leafing is predicted to precede birch leafing in 92% of years. We can also compare the Marsham record with first leafing records for silver birch and pedunculate oak collected by citizen scientists in the same region for the period 1999–2011. While silver birch preceded oak each year during the recent period (Fig. 4), the average annual difference in mean first leafing dates was 11.41 days (± 0.46) down from 18.23 (± 1.03) in the historic time series. In broad support of our predictions the two years preceded by the warmest autumn/winter, 2000 and 2007 had the smallest difference in leafing times, with silver birch coming into leaf just two days earlier than pedunculate oak in 2000.

## Discussion

In agreement with earlier work on the Marsham dataset (Sparks & Carey, 1995), we find that spring forcing plays a strong role in determining the phenology of all species, and a subset of these species are also sensitive to cold temperatures during the preceding year. A positive slope for the regression of phenology on autumn temperatures has been reported by several correlative analyses of plant time series (Fitter *et al*., 1995; Cook *et al*., 2012). It is certainly possible that the slopes identified by ourselves and others reflect chilling requirements, as the UniChill model assumes (Chuine, 2000), and there is ample experimental evidence that a period of winter chilling brings budburst of high‐latitude deciduous trees forward (Laube *et al*., 2014).

The early timing of the period of chilling sensitivity may, however, be consistent with an alternative mechanism of thermal sensitivity, where warm autumn conditions delay the opportunity for bud dormancy induction, which has been shown experimentally in boreal trees (Heide, 2003). Delayed dormancy could in turn delay the period of chilling unit accumulation. If this were true, the UniChill model chilling function (Chuine, 2000) may be detecting a signature of dormancy induction, meaning that future modeling would benefit from further parameters to capture this additional process. Fitting such a model will be challenging and might require other parameters to be fixed based on experimental insights. A signature of dormancy induction would also resolve an apparent disagreement between our results and the experimental finding of Laube *et al*. (2014). They report that the low chilling requirements of pioneer tree species, such as birch, mean that they are relatively less impacted by warm winters than canopy tree species, such as beech, but as their experiments took place after dormancy induction they may have missed a signature that we detect.

As a consequence of interspecific variation in sensitivities to forcing and chilling we predict a substantial re‐ordering of forest phenology under future climate, the ecological consequences of which are not currently known. It seems likely, however, that earlier shading by canopy trees will impact negatively on the growth of trees in the understorey and recruitment of their seedlings (Laube *et al*., 2014). Tree‐rings represent a source of information on the impacts of past conditions on growth (Čufar *et al*., 2008); therefore, it might be possible to model the phenology of multiple coexisting species into the past on the basis of historic temperature data and to use these predictions to test the impact of relative phenology upon growth (although controlling for confounding environmental influences would necessitate a long‐time series and detailed knowledge of woodland management). If warmer winters cause the early year growth of species with historically late phenology (e.g., oak) to impact negatively on the early year growth of species with historically early phenology (e.g., birch), then this may lead to strong selection for earlier leafing in the latter. At the same time those species projected to advance the most may face greater damage from late frosts (Polgar & Primack, 2011). The net effect that these factors will have on forest communities is unknown, although we suggest that shifts in the abundance of species and community composition will be a more likely long‐term outcome than genetic adaptation of species (De Mazancourt *et al*., 2008). In recent years mechanistic models that link phenological responses to species distributions have been developed (Chuine & Beaubien, 2001; Morin *et al*., 2007), and a next step would be to develop models to test whether the relative phenology of interacting species leaves a detectable imprint on species distributions.

On a methodological note, the temporal replication and free availability of the Marsham series (Margary, 1926; Sparks & Carey, 1995) make it well suited as a benchmark dataset for phenology. We have reported several statistics that pertain to model explanatory power (R^2^, root mean square error, and AIC) and against which the performance of novel parameter‐rich models might usefully be compared.

Taken together, we find that the spring phenology of each of the focal forest species is highly sensitive to spring temperatures, but that species vary substantially in their sensitivity to winter and spring temperatures. Our projections reveal that this may lead to a substantial shuffling of the order of flowering and leafing events in temperate forests. Identifying the fitness and ecological consequences of such shifts in the relative phenology of interacting species should be a priority for future work addressing climate impacts.

## Supporting information


**Figure S1** Maximum likelihood chilling and forcing functions in relation to temperature under the preferred mechanistic model for each species. Note that the models for three species have no chilling requirement.

**Table S1** Coefficients of determination, *R* ^2^, for models fitted, with summary of parameters estimated for (a) regression models and (b) mechanistic models.
**Table S2** The relative proportion of years when the phenology of species A (rows) precedes the phenology of species B (columns) in (a) the historic data, and predicted data for (b) 2010–2039 and (c) 2040–2069.
