# Supplementary figures and images for: Predicting a change in the order of spring phenology in temperate forests
Source: Glob Chang Biol. 2015 Mar 2;21(7):2603–11. doi: 10.1111/gcb.12896 (PMC4964954; doi:10.1111/gcb.12896)

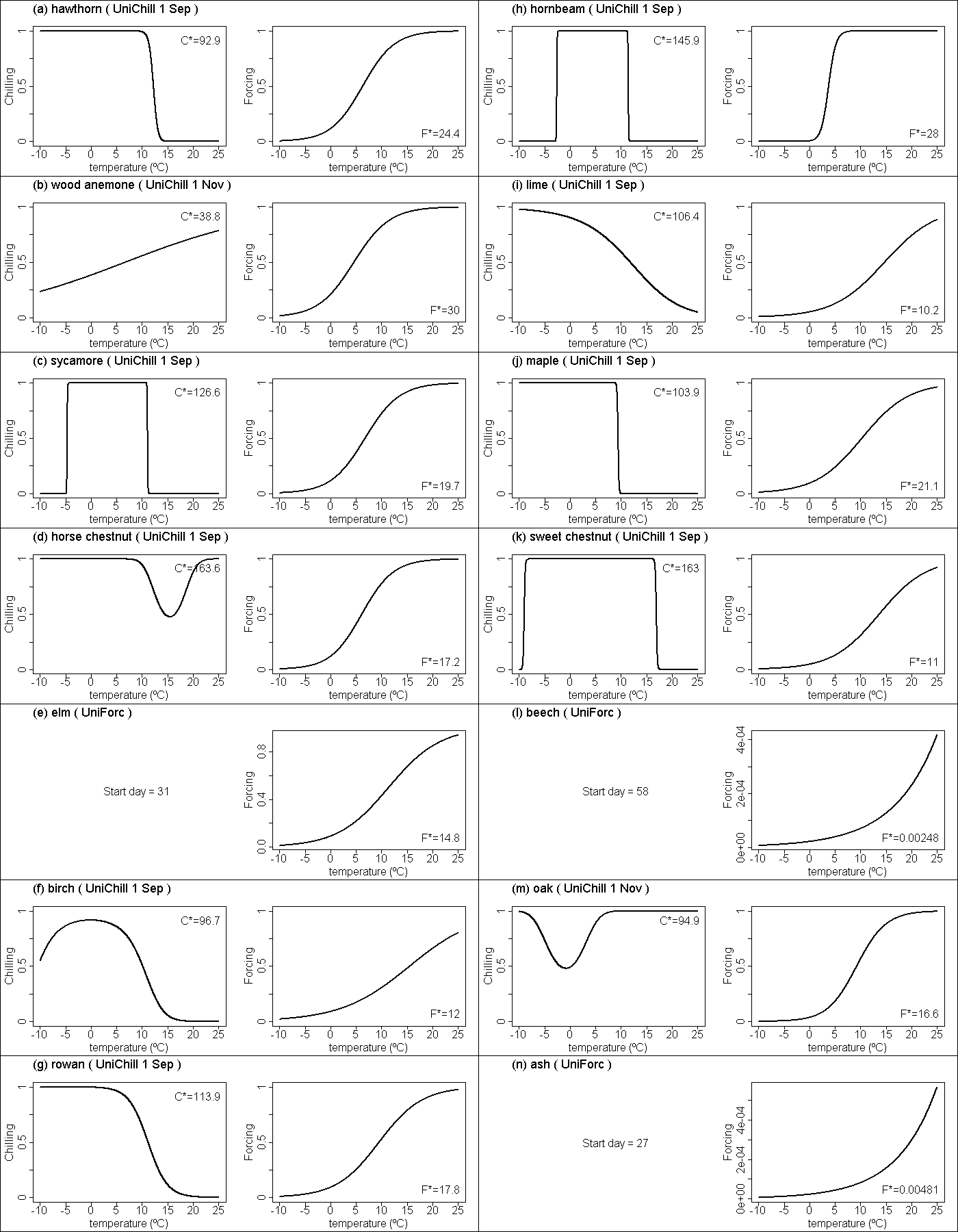

Supplement: Supplementary file 1 — Figure S1 Maximum likelihood chilling and forcing functions in relation to temperature under the preferred mechanistic model for each species. Note that the models for three species have no chilling requirement. [file GCB-21-2603-s001.tiff]
