# Supplementary material for: Predicting a change in the order of spring phenology in temperate forests
Source: Glob Chang Biol. 2015 Mar 2;21(7):2603–11. doi: 10.1111/gcb.12896 (PMC4964954; doi:10.1111/gcb.12896)
Supplement: Supplementary file 2 — Table S1 Coefficients of determination, R 2, for models fitted, with summary of parameters estimated for (a) regression models and (b) mechanistic models. Table S2 The relative proportion of years when the phenology of species A (rows) precedes the phenology of species B (columns) in (a) the historic data, and predicted data for (b) 2010–2039 and (c) 2040–2069. [file GCB-21-2603-s002.docx]

Table S1: Parameter estimates and summary statistics for (a) single time-window, (b) double time-window, (c) PSR, (d) uniforc, (e) unichill (September start) and (f) unichill (November start) models

(a)

| Species | R^2^ | Window start and end (ordinal dates) | Intercept  +/- se | Slope  +/- se  (days/°C) |  |
| --- | --- | --- | --- | --- | --- |
| hawthorn | 0.48 | 3 to 75 | 104.01 +/- 3.41 | -9.33 +/- 0.82 |  |
| wood anemone | 0.5 | -4 to 105 | 119.84 +/- 3.17 | -7.85 +/- 0.67 |  |
| sycamore | 0.38 | 46 to 102 | 126.34 +/- 4.05 | -6.54 +/- 0.73 |  |
| horse chestnut | 0.48 | 52 to 95 | 121.84 +/- 2.54 | -5.32 +/- 0.47 |  |
| elm | 0.31 | 32 to 92 | 122.62 +/- 3.95 | -5.79 +/- 0.8 |  |
| birch | 0.4 | 40 to 84 | 120.99 +/- 2.77 | -5.53 +/- 0.57 |  |
| rowan | 0.57 | 32 to 97 | 127.28 +/- 2.41 | -6.34 +/- 0.47 |  |
| hornbeam | 0.28 | 33 to 90 | 124.28 +/- 3.84 | -5.7 +/- 0.78 |  |
| lime | 0.53 | 40 to 128 | 155.22 +/- 4.24 | -8.35 +/- 0.66 |  |
| maple | 0.29 | 40 to 139 | 163.45 +/- 8.9 | -7.98 +/- 1.28 |  |
| sweet chestnut | 0.52 | 43 to 104 | 143.18 +/- 2.98 | -6.39 +/- 0.54 |  |
| beech | 0.46 | 60 to 123 | 143.71 +/- 3.13 | -5.06 +/- 0.46 |  |
| oak | 0.64 | 56 to 126 | 171.33 +/- 3.72 | -8.68 +/- 0.55 |  |
| ash | 0.32 | 27 to 128 | 155.4 +/- 4.78 | -6.07 +/- 0.78 |  |

(b)

| Species | R^2^ | First window start and end (ordinal dates) | Intercept  +/- se | First window Slope  +/- se  (days/°C) | Second window Slope  +/- se  (days/°C) |
| --- | --- | --- | --- | --- | --- |
| hawthorn | 0.48 | -140 to -32 | 24.5 +/- 17.35 | 7.36 +/- 1.58 | -8.92 +/- 0.77 |
| wood anemone | 0.55 | -150 to -136 | 91.7 +/- 9.7 | 1.82 +/- 0.6 | -8.07 +/- 0.65 |
| sycamore | 0.53 | -151 to -63 | 70.44 +/- 14.9 | 4.38 +/- 1.13 | -6.64 +/- 0.69 |
| horse chestnut | 0.44 | -135 to -46 | 88.47 +/- 9.43 | 2.97 +/- 0.81 | -5.27 +/- 0.45 |
| elm | 0.52 | -98 to -73 | 99.65 +/- 10.38 | 2.06 +/- 0.86 | -5.63 +/- 0.79 |
| birch | 0.34 | -139 to -50 | 57.93 +/- 12.56 | 5.39 +/- 1.05 | -5.38 +/- 0.53 |
| rowan | 0.5 | -103 to -22 | 95 +/- 5.96 | 3.91 +/- 0.67 | -6.15 +/- 0.42 |
| hornbeam | 0.66 | -128 to -80 | 84.33 +/- 15.94 | 3.03 +/- 1.17 | -5.47 +/- 0.77 |
| lime | 0.32 | -103 to -11 | 131.5 +/- 6.5 | 3.3 +/- 0.72 | -8.53 +/- 0.62 |
| maple | 0.6 | -84 to -73 | 130.62 +/- 11.18 | 2.91 +/- 0.68 | -7.43 +/- 1.18 |
| sweet chestnut | 0.41 | -214 to -192 | 117.21 +/- 7.7 | 1.96 +/- 0.54 | -6.66 +/- 0.52 |
| beech | 0.56 | -30 to -16 | 142.45 +/- 3.14 | 0.48 +/- 0.22 | -5.18 +/- 0.46 |
| oak | 0.48 | -10 to 45 | 172.85 +/- 3.47 | -1.55 +/- 0.32 | -8.1 +/- 0.52 |
| ash | 0.69 | -195 to -186 | 135.2 +/- 9.71 | 1.31 +/- 0.55 | -5.96 +/- 0.77 |

(c)

| Species | R^2^ | Chilling duration (days) | Chilling effect (days/°C) | Forcing duration (days) | Forcing effect (days/°C) |
| --- | --- | --- | --- | --- | --- |
| hawthorn | 0.56 | 96 | 5.89 | 100 | -9.25 |
| wood anemone | 0.56 | 38 | 1.22 | 161 | -8.67 |
| sycamore | 0.43 | 43 | 1.79 | 78 | -6.05 |
| horse chestnut | 0.50 | 49 | 1.62 | 83 | -5.39 |
| elm | 0.33 | 24 | 0.86 | 84 | -5.98 |
| birch | 0.51 | 101 | 4.97 | 67 | -5.88 |
| rowan | 0.67 | 89 | 4.02 | 91 | -7.23 |
| hornbeam | 0.31 | 33 | 1.04 | 122 | -7.29 |
| lime | 0.60 | 87 | 2.51 | 108 | -8.47 |
| maple | 0.34 | 81 | 2.50 | 120 | -7.30 |
| sweet chestnut | 0.56 | 27 | 0.99 | 104 | -7.19 |
| beech | 0.46 | 0 | 0.00 | 101 | -5.04 |
| oak | 0.73 | 63 | 1.63 | 167 | -9.18 |
| ash | 0.32 | 0 | 0.00 | 126 | -6.51 |

(d)

| Species | R^2^ | t_0_ | b_f_ | c_f_ | F^*^ | Root Mean Square Error (RMSE) |
| --- | --- | --- | --- | --- | --- | --- |
| hawthorn | 0.51 | 4 | -0.38 | 4.77 | 27.716 | 13.31 |
| wood anemone | 0.49 | 18 | -0.36 | 3.09 | 39.092 | 9.23 |
| sycamore | 0.38 | 20 | -0.13 | 37.17 | 0.953 | 10.44 |
| horse chestnut | 0.48 | 33 | -0.12 | 70.85 | 0.033 | 7.48 |
| elm | 0.33 | 31 | -0.20 | 11.40 | 14.826 | 12.15 |
| birch | 0.42 | 22 | -0.13 | 68.01 | 0.023 | 9.81 |
| rowan | 0.62 | 31 | -0.24 | 9.50 | 17.832 | 6.97 |
| hornbeam | 0.29 | 33 | -0.29 | 4.84 | 33.152 | 12.74 |
| lime | 0.58 | 26 | -0.16 | 60.17 | 0.011 | 7.53 |
| maple | 0.27 | 23 | -0.17 | 11.95 | 22.354 | 11.37 |
| sweet chestnut | 0.58 | 42 | -0.28 | 9.27 | 19.533 | 7.28 |
| beech | 0.50 | 58 | -0.12 | 90.57 | 0.002 | 5.52 |
| oak | 0.73 | 26 | -0.20 | 20.56 | 4.615 | 5.52 |
| ash | 0.37 | 27 | -0.13 | 83.00 | 0.005 | 8.79 |

(e)

| Species | R^2^ | a_c_ | b_c_ | c_c_ | C^*^ | b_f_ | c_f_ | F* | Mean ordinal date on which C* satisfied | Standard deviation of ordinal date on which C* satisfied | Root Mean Square Error (RMSE) |
| --- | --- | --- | --- | --- | --- | --- | --- | --- | --- | --- | --- |
| hawthorn | 0.60 | 0.08 | 2.33 | 12.26 | 92.88 | -0.32 | 6.28 | 24.37 | -4.23 | 6.34 | 11.99 |
| wood anemone | 0.54 | 0.00 | -0.25 | -4.89 | 134.03 | -0.30 | 4.59 | 30.00 | 22.40 | 3.35 | 8.77 |
| sycamore | 0.43 | 1.55 | -24.45 | -4.68 | 126.60 | -0.30 | 6.76 | 19.72 | 39.56 | 7.30 | 10.00 |
| horse chestnut | 0.53 | -0.10 | 0.19 | 14.50 | 163.63 | -0.32 | 6.12 | 17.22 | 55.35 | 2.90 | 7.16 |
| elm | 0.36 | 0.00 | 0.25 | 14.96 | 124.42 | -0.20 | 11.09 | 15.36 | 30.33 | 3.56 | 11.87 |
| birch | 0.51 | 0.02 | 0.47 | 10.15 | 96.73 | -0.15 | 15.47 | 12.04 | 28.56 | 6.36 | 9.01 |
| rowan | 0.70 | 0.00 | 0.53 | 11.19 | 113.95 | -0.24 | 9.49 | 17.85 | 30.03 | 5.81 | 6.23 |
| hornbeam | 0.36 | 1.63 | -22.88 | -2.59 | 145.92 | -1.38 | 3.74 | 27.98 | 58.42 | 8.77 | 12.15 |
| lime | 0.62 | 0.00 | 0.21 | 11.81 | 106.41 | -0.20 | 14.69 | 10.20 | 33.42 | 4.87 | 7.10 |
| maple | 0.36 | 0.44 | 11.18 | 9.43 | 103.93 | -0.22 | 10.26 | 21.06 | 32.18 | 8.94 | 10.64 |
| sweet chestnut | 0.60 | 0.38 | 9.81 | 16.95 | 162.97 | -0.22 | 13.64 | 11.02 | 42.50 | 3.04 | 7.09 |
| beech | 0.51 | 0.14 | 5.25 | 19.24 | 179.00 | -0.12 | 59.85 | 0.09 | 56.91 | 0.79 | 5.42 |
| oak | 0.77 | -0.13 | 0.11 | -0.27 | 160.33 | -0.41 | 8.00 | 20.12 | 58.99 | 7.15 | 5.09 |
| ash | 0.38 | 0.17 | 4.13 | 18.52 | 172.49 | -0.17 | 59.27 | 0.01 | 50.84 | 1.47 | 8.74 |

(f)

| Species | R^2^ | a_c_ | b_c_ | c_c_ | C^*^ | b_f_ | c_f_ | F* | Mean ordinal date on which C* satisfied | Standard deviation of ordinal date on which C* satisfied | Root Mean Square Error (RMSE) |
| --- | --- | --- | --- | --- | --- | --- | --- | --- | --- | --- | --- |
| hawthorn | 0.52 | 1.57 | -24.09 | -4.43 | 61.43 | -0.43 | 3.81 | 32.52 | 1.80 | 2.02 | 13.11 |
| wood anemone | 0.55 | 0.00 | -0.07 | 6.67 | 38.80 | -0.29 | 4.57 | 30.03 | 22.69 | 3.95 | 8.68 |
| sycamore | 0.39 | 0.40 | 8.30 | 15.87 | 80.87 | -0.13 | 37.29 | 1.06 | 19.59 | 1.53 | 10.40 |
| horse chestnut | 0.50 | 0.51 | 13.50 | 10.98 | 111.91 | -0.34 | 6.18 | 18.39 | 51.63 | 1.94 | 7.32 |
| elm | 0.36 | 2.35 | -28.08 | -2.86 | 94.02 | -0.38 | 5.86 | 23.30 | 42.81 | 5.89 | 11.86 |
| birch | 0.44 | 0.00 | 0.10 | 14.97 | 59.39 | -0.17 | 12.97 | 15.53 | 19.35 | 2.35 | 9.67 |
| rowan | 0.66 | 0.18 | 14.87 | 10.31 | 90.22 | -0.26 | 8.71 | 19.07 | 31.51 | 2.57 | 6.58 |
| hornbeam | 0.34 | 1.90 | -22.67 | -1.57 | 92.11 | -0.30 | 5.33 | 28.62 | 38.68 | 6.50 | 12.31 |
| lime | 0.62 | 0.30 | 16.55 | 9.88 | 88.90 | -0.22 | 10.24 | 18.95 | 31.28 | 3.53 | 7.18 |
| maple | 0.31 | 0.36 | 14.93 | 9.69 | 86.76 | -0.27 | 7.25 | 31.13 | 29.93 | 3.74 | 11.05 |
| sweet chestnut | 0.59 | 0.32 | 6.35 | 11.41 | 101.80 | -0.27 | 9.36 | 19.60 | 41.09 | 1.42 | 7.22 |
| beech | 0.50 | 0.02 | 6.08 | 13.33 | 118.82 | -0.12 | 79.56 | 0.01 | 57.13 | 0.52 | 5.49 |
| oak | 0.77 | -0.06 | 0.14 | -1.93 | 94.94 | -0.35 | 9.22 | 16.63 | 58.15 | 7.89 | 5.04 |
| ash | 0.39 | 0.95 | 14.63 | 10.65 | 110.00 | -0.20 | 16.44 | 8.97 | 51.52 | 2.68 | 8.62 |

^*^ For PSR, the periods of chilling and forcing are defined as those days whose estimated effect is +/- standard errors of zero. The chilling or forcing effect is the sum of the regression coefficients in the identified time period.

Table S2. The relative proportion of years when the phenology of species A (rows) precedes the phenology of species B (columns) in (a) the historic data, and predicted data for (b) 2010-2039 and (c) 2040-2069.

(a)

|  | hawthorn | wood anemone | sycamore | horse chestnut | elm | birch | rowan | hornbeam | lime | maple | sweet chestnut | beech | oak | ash |
| --- | --- | --- | --- | --- | --- | --- | --- | --- | --- | --- | --- | --- | --- | --- |
| hawthorn | - | 0.824 | 0.954 | 0.962 | 0.941 | 0.968 | 0.994 | 0.98 | 0.994 | 0.99 | 1 | 1 | 1 | 1 |
| wood anemone | 0.148 | - | 0.716 | 0.824 | 0.773 | 0.791 | 0.825 | 0.815 | 0.957 | 0.928 | 0.985 | 0.993 | 1 | 1 |
| sycamore | 0.026 | 0.269 | - | 0.53 | 0.553 | 0.514 | 0.639 | 0.676 | 0.858 | 0.951 | 0.964 | 0.953 | 0.98 | 1 |
| horse chestnut | 0.032 | 0.155 | 0.362 | - | 0.478 | 0.57 | 0.57 | 0.664 | 0.856 | 0.942 | 0.958 | 0.968 | 0.987 | 0.993 |
| elm | 0.037 | 0.202 | 0.402 | 0.478 | - | 0.454 | 0.477 | 0.646 | 0.689 | 0.865 | 0.831 | 0.902 | 0.904 | 0.962 |
| birch | 0.019 | 0.187 | 0.405 | 0.364 | 0.485 | - | 0.45 | 0.5 | 0.745 | 0.902 | 0.879 | 0.903 | 0.935 | 0.979 |
| rowan | 0 | 0.139 | 0.286 | 0.305 | 0.432 | 0.376 | - | 0.607 | 0.779 | 0.911 | 0.894 | 0.921 | 0.974 | 0.986 |
| hornbeam | 0.007 | 0.178 | 0.261 | 0.282 | 0.291 | 0.432 | 0.338 | - | 0.549 | 0.853 | 0.761 | 0.808 | 0.863 | 0.978 |
| lime | 0.006 | 0.043 | 0.095 | 0.098 | 0.273 | 0.208 | 0.154 | 0.396 | - | 0.631 | 0.755 | 0.796 | 0.845 | 0.972 |
| maple | 0.01 | 0.072 | 0.049 | 0.058 | 0.104 | 0.069 | 0.04 | 0.088 | 0.262 | - | 0.556 | 0.51 | 0.625 | 0.812 |
| sweet chestnut | 0 | 0.015 | 0.022 | 0.035 | 0.121 | 0.093 | 0.056 | 0.203 | 0.175 | 0.444 | - | 0.479 | 0.646 | 0.83 |
| beech | 0 | 0 | 0.033 | 0.026 | 0.09 | 0.078 | 0.053 | 0.151 | 0.164 | 0.394 | 0.415 | - | 0.62 | 0.804 |
| oak | 0 | 0 | 0.02 | 0.006 | 0.081 | 0.039 | 0.013 | 0.082 | 0.084 | 0.26 | 0.257 | 0.272 | - | 0.726 |
| ash | 0 | 0 | 0 | 0 | 0.015 | 0.014 | 0.007 | 0.007 | 0.014 | 0.139 | 0.133 | 0.14 | 0.226 | - |

(b)

|  | hawthorn | wood anemone | sycamore | horse chestnut | elm | birch | rowan | hornbeam | lime | maple | sweet chestnut | beech | oak | ash |
| --- | --- | --- | --- | --- | --- | --- | --- | --- | --- | --- | --- | --- | --- | --- |
| hawthorn | - | 0.14 | 0.954 | 0.925 | 0.859 | 0.997 | 0.995 | 0.968 | 0.972 | 0.999 | 0.99 | 0.995 | 0.968 | 0.999 |
| wood anemone | 0.86 | - | 0.998 | 0.993 | 0.996 | 1 | 1 | 1 | 1 | 1 | 1 | 1 | 1 | 1 |
| sycamore | 0.046 | 0.002 | - | 0.197 | 0.165 | 0.845 | 0.72 | 0.679 | 0.487 | 0.986 | 0.718 | 0.748 | 0.609 | 0.981 |
| horse chestnut | 0.075 | 0.007 | 0.803 | - | 0.252 | 0.983 | 0.96 | 0.786 | 0.828 | 0.999 | 0.934 | 0.976 | 0.841 | 1 |
| elm | 0.141 | 0.004 | 0.835 | 0.748 | - | 0.995 | 0.989 | 0.905 | 0.971 | 1 | 1 | 1 | 0.985 | 1 |
| birch | 0.003 | 0 | 0.155 | 0.017 | 0.005 | - | 0.269 | 0.45 | 0.209 | 0.98 | 0.512 | 0.515 | 0.381 | 0.971 |
| rowan | 0.005 | 0 | 0.28 | 0.04 | 0.011 | 0.731 | - | 0.548 | 0.303 | 0.989 | 0.653 | 0.662 | 0.488 | 0.988 |
| hornbeam | 0.032 | 0 | 0.321 | 0.214 | 0.095 | 0.55 | 0.452 | - | 0.406 | 0.902 | 0.586 | 0.594 | 0.502 | 0.966 |
| lime | 0.028 | 0 | 0.513 | 0.172 | 0.029 | 0.791 | 0.697 | 0.594 | - | 0.998 | 0.849 | 0.849 | 0.699 | 1 |
| maple | 0.001 | 0 | 0.014 | 0.001 | 0 | 0.019 | 0.011 | 0.098 | 0.002 | - | 0.045 | 0.05 | 0.035 | 0.848 |
| sweet chestnut | 0.01 | 0 | 0.282 | 0.066 | 0 | 0.488 | 0.347 | 0.414 | 0.151 | 0.955 | - | 0.569 | 0.284 | 0.999 |
| beech | 0.005 | 0 | 0.252 | 0.024 | 0 | 0.485 | 0.338 | 0.406 | 0.151 | 0.95 | 0.431 | - | 0.29 | 1 |
| oak | 0.032 | 0 | 0.391 | 0.159 | 0.015 | 0.619 | 0.512 | 0.498 | 0.301 | 0.965 | 0.716 | 0.71 | - | 1 |
| ash | 0.001 | 0 | 0.019 | 0 | 0 | 0.029 | 0.012 | 0.034 | 0 | 0.152 | 0.001 | 0 | 0 | - |

(c)

|  | hawthorn | wood anemone | sycamore | horse chestnut | elm | birch | rowan | hornbeam | lime | maple | sweet chestnut | beech | oak | ash |
| --- | --- | --- | --- | --- | --- | --- | --- | --- | --- | --- | --- | --- | --- | --- |
| hawthorn | - | 0.14 | 0.944 | 0.864 | 0.67 | 1 | 1 | 0.946 | 0.889 | 0.999 | 0.987 | 0.982 | 0.859 | 0.998 |
| wood anemone | 0.86 | - | 0.998 | 0.991 | 0.992 | 1 | 1 | 0.999 | 0.998 | 1 | 1 | 1 | 0.998 | 1 |
| sycamore | 0.056 | 0.002 | - | 0.127 | 0.099 | 0.68 | 0.441 | 0.59 | 0.177 | 0.854 | 0.388 | 0.348 | 0.228 | 0.795 |
| horse chestnut | 0.136 | 0.009 | 0.873 | - | 0.155 | 0.995 | 0.983 | 0.794 | 0.573 | 0.998 | 0.872 | 0.915 | 0.569 | 0.997 |
| elm | 0.33 | 0.008 | 0.901 | 0.845 | - | 0.999 | 0.998 | 0.942 | 0.911 | 0.999 | 0.999 | 1 | 0.913 | 1 |
| birch | 0 | 0 | 0.281 | 0.005 | 0.001 | - | 0.205 | 0.48 | 0.033 | 0.859 | 0.215 | 0.139 | 0.077 | 0.745 |
| rowan | 0 | 0 | 0.536 | 0.017 | 0.002 | 0.775 | - | 0.594 | 0.061 | 0.927 | 0.345 | 0.27 | 0.118 | 0.851 |
| hornbeam | 0.053 | 0.001 | 0.383 | 0.206 | 0.058 | 0.499 | 0.393 | - | 0.242 | 0.756 | 0.369 | 0.358 | 0.249 | 0.746 |
| lime | 0.111 | 0.002 | 0.823 | 0.427 | 0.089 | 0.967 | 0.939 | 0.758 | - | 0.998 | 0.886 | 0.915 | 0.538 | 0.999 |
| maple | 0.001 | 0 | 0.099 | 0.002 | 0.001 | 0.094 | 0.052 | 0.215 | 0.002 | - | 0.044 | 0.018 | 0.009 | 0.524 |
| sweet chestnut | 0.013 | 0 | 0.612 | 0.128 | 0.001 | 0.785 | 0.655 | 0.631 | 0.114 | 0.956 | - | 0.427 | 0.069 | 0.992 |
| beech | 0.018 | 0 | 0.652 | 0.085 | 0 | 0.861 | 0.73 | 0.642 | 0.085 | 0.982 | 0.573 | - | 0.106 | 1 |
| oak | 0.141 | 0.002 | 0.772 | 0.431 | 0.087 | 0.923 | 0.882 | 0.751 | 0.462 | 0.991 | 0.931 | 0.894 | - | 1 |
| ash | 0.002 | 0 | 0.205 | 0.003 | 0 | 0.255 | 0.149 | 0.254 | 0.001 | 0.476 | 0.008 | 0 | 0 | - |

Predictions that result in no event are treated as occurring later than those where an event was predicted.
